# Nurses’ Perceptions of Climate Change: Protocol for a Scoping Review

**DOI:** 10.2196/42516

**Published:** 2023-01-11

**Authors:** Thierno Diallo, Anouk Bérubé, Martin Roberge, Pierre-Paul Audate, Stéphanie Larente-Marcotte, Édith Jobin, Nisrine Moubarak, Laurence Guillaumie, Sophie Dupéré, Anne Guichard, Isabelle Goupil-Sormany

**Affiliations:** 1 Faculty of Nursing Sciences Laval University Quebec, QC Canada; 2 VITAM - Centre de Recherche en Santé Durable Quebec, QC Canada; 3 Centre hospitalier universitaire de Québec–Université Laval Research Centre Quebec, QC Canada; 4 Institut National de Santé Publique du Québec Quebec, QC Canada; 5 Centre de Recherche en Aménagement et Développement Laval University Quebec, QC Canada; 6 Centre de Recherche de Montréal sur les Inégalités Sociales, les Discriminations et les Pratiques Alternatives de Citoyenneté Montreal, QC Canada; 7 Department of Social and Preventive Medicine Faculty of Medicine Laval University Quebec, QC Canada

**Keywords:** perception, climate change, nurse, scoping review, review method, environment, nursing, perspective, search strategy, global warming, health care professional

## Abstract

**Background:**

Climate change is a major threat to human health. Nurses are in contact with patients suffering from the effects of climate change in their daily work. Therefore, they need to be involved in combating it at both the individual and collective levels. However, there is still very little known about nurses’ perception of climate change and their role toward it. A few recent studies have embarked on the process of examining the perceptions of these health professionals relative to climate change, but no exploratory review of the literature has been conducted on nurses’ perception of this phenomenon.

**Objective:**

The purpose of this protocol is to develop a research strategy for an exploratory review of the literature focused on identifying nurses’ perceptions of climate change.

**Methods:**

Firstly, with the help of a specialized librarian, we defined keywords and their combinations, using an iterative process, to develop a documentary search strategy. This strategy was tested in the following four bibliographic databases: MEDLINE (PubMed), CINAHL, Embase, and Web of Science. A search of the grey literature will also be conducted to supplement the results of the bibliographic database search. The next step will be for 2 members of the research team to carry out a 2-stage selection process using the web-based systematic review software Covidence. They will carry out this selection process independently, with the aim of identifying relevant studies that meet the inclusion criteria for our exploratory review. Finally, data on year of publication, authors, geographic area, article type, study objectives, methodology, and key findings will be extracted from selected articles for analysis. The data will be analyzed by the research team based on an in-depth examination of the findings and will be directed toward answering the research question and fulfilling the study’s objective.

**Results:**

The results will help in defining nurses’ perceptions of climate change more clearly as well as the role they can play and what they need to be able to bring forward solutions to this phenomenon. The findings should also serve to guide the health sector and nursing faculty’s interventions aimed at preparing health professionals to act on the potential threats associated with climate change.

**Conclusions:**

The preliminary search suggests a possible gap between the importance of the nursing role in addressing the health impacts of climate change and the nurses’ lack of knowledge and awareness on this matter. The results will allow for raising nurses’ awareness of their role in the fight against climate change and the ways to address its health effects. This study will also open up new research perspectives on how to equip nurses to better integrate response to climate change issues into their professional practice.

**International Registered Report Identifier (IRRID):**

DERR1-10.2196/42516

## Introduction

Climate change represents one of the greatest threats to human health in the 21st century [1-4]. The new report from the Intergovernmental Panel on Climate Change indicates that climate change is already affecting the physical health and well-being of many people around the world [5]. This is reaffirmed by a recent Health Canada report, which states that climate change is already having a negative impact on the health of Canadians due to increased temperatures, extreme heat, forest fires, and the geographic spread of zoonotic diseases such as Lyme disease [6]. Climate change affects the physical and mental health of populations in direct and indirect ways. Examples of the direct health effects of climate change include extreme weather events, such as storms, wildfires, floods, or drought; increased exposure to pollens; and heat waves [7]. Moreover, a 2011 study published in the journal *Science* found that the frequency of such events is expected to increase by a factor of 5 to 10 over the next 40 years [8]. Examples of indirect effects include air pollution (causing respiratory and heart diseases), water pollution (causing gastroenteritis), changes in ecosystems and in the geographic distribution of vector-borne diseases, food insecurity, population displacement, conflict, and mental health [7,9-12]. The World Health Organization estimates that climate change could cause approximately 250,000 additional deaths per year between 2030 and 2050 due to malnutrition, malaria, diarrhea, and heat stress [13].

Health professionals have an important role to play in combating health threats related to climate change [14]. Nurses, who make up a significant portion of health professionals engaged in improving the health and well-being of individuals [15-17], have a critical role to play in responding to and reducing health issues stemming from climate change [18]. Because they benefit from a high degree of public trust [19], nurses can play a leadership role in the health sector both by increasing awareness of climate change as an important health issue and by spearheading action and advocacy in the fight against climate change [20]. In their daily work, nurses also have an advocacy role toward their patients experiencing the effects of climate change, especially those who are the most vulnerable. Therefore, nurses need to be involved in combating climate change at both the individual and collective levels. However, there is still very little known about nurses’ perception of climate change and of their role toward it. A few recent studies have examined nurses’ perceptions of climate change and health [16,17,21-23]. In addition, two exploratory reviews of the literature have been conducted. The first, conducted in 2018, focused on health professionals’ knowledge, attitudes, perceptions, and practices related to climate change and its health impacts [14]. The second, conducted in 2022, concerns awareness and attitudes among nursing students and educators related to sustainability and climate change [24]. To the best of our knowledge, no study has yet been conducted for the purpose of identifying and synthesizing nurses’ perceptions of climate change in an exploratory literature review. The 2018 study considers health care professionals in general, and the 2022 study focuses on nursing students and educators.

The purpose of this protocol is to develop a research strategy for an exploratory literature review aimed at identifying nurses’ perceptions of climate change.

## Methods

### Overview

The exploratory literature review will be conducted in 5 steps, as described by Arksey and O’Malley [25] and refined by Levac et al [26]. These steps are as follows:

1. Identification of the research question
2. Identification of relevant studies
3. Selection of relevant and reliable studies
4. Charting the data from the included studies
5. Collating, summarizing, and reporting the findings

### Identification of the Research Question

As recommended by Levac et al [26], the question we wish to answer through this exploratory review has been clearly articulated and focused. The question under study in this exploratory literature review is, “what are nurses’ perceptions relative to climate change?” This question is justified by the need to advance knowledge about nurses’ perceptions of climate change to better prepare them to tackle these climate issues [16,20,22,23].

### Identification of Relevant Studies

We first developed a search strategy with the help of a specialized librarian using an iterative process. Searches were conducted in the following four bibliographic databases targeting biomedical and interdisciplinary topics: MEDLINE (PubMed), CINAHL, Embase, and Web of Science. Two concepts were used to identify keywords for the literature search strategy: climate change (concept 1) and nurses’ perceptions (concept 2). Once identified, the keywords were classified into free-text search terms and controlled vocabulary to define a search strategy specific to each database. The characteristics of free-text and controlled vocabulary searches in the databases used are presented in Table 1.

Table 2 presents the keywords (free-text search terms) and thesauri terms (controlled vocabulary) for each of the four databases for each of the two concepts defined previously.

An example of a preliminary MEDLINE (PubMed) search strategy is shown in Table 3.

**Table 1:** Free-text and controlled vocabulary search characteristics for databases used for this study.

Selected databases	Search vocabulary
MEDLINE via PubMed	Free text: title/abstract or tiab (advanced) Controlled vocabulary: MeSHa
CINAHL Plus with full text (EBSCO)	Free text: title + abstract, ti+ab (two separate searches) Controlled vocabulary: CINAHL descriptors
Embase	Free text: title, abstract, keyword, ti,ab,kw (quick search) Controlled vocabulary: EMTREE
Web of Science	Free text only: topic (includes title, abstract, author keywords, and Keywords Plus).

^a^MeSH: Medical Subject Headings.

**Table 2:** Free-text search terms (keywords) and controlled vocabulary.

Concepts and free-text search terms (Keywords)			Controlled vocabulary (thesaurus terms in electronic bibliographic databases)					
			MEDLINE (PubMed)			CINAHL Plus with Full Text (EBSCO)		Embase
**Concept 1: climate change**								
	“Climate change*” OR “global warming” OR “climate crisis” OR “climate warming” OR “climate issue*” OR “Climate vulnerabilit*” OR “Climate Emergenc*” OR “Greenhouse Effect” OR “climate action”	“Climate Change”[Mesh^a^] OR “Greenhouse Effect”[Mesh]		(MH “Climate Change”) OR (MH “Greenhouse Effect”)			'Climate change'/exp OR 'greenhouse effect'/de	
**Concept 2: nurses’ perceptions**								
	(Nurse OR nurses OR nursing) AND (attitude* OR perception* OR awareness OR perspective* OR belief* OR views OR behavior* OR opinion* OR concern* OR understanding OR knowledge*)	(“Nurses”[Mesh]) OR “Nurse’s Role”[Mesh]) AND (“Perception”[Mesh:NoExp] OR “Attitude”[Mesh:NoExp] OR “Attitude of Health Personnel”[Mesh:NoExp] OR “Awareness”[Mesh] OR “Knowledge”[Mesh] OR “Behavior”[Mesh:NoExp])			(MH “Nurse Attitudes”) OR ((MH “Nurses+”) OR (MH “Nursing Role”)) AND ((MH “Attitude”) OR (MH “Perception”) OR (MH “Knowledge”) OR (MH “Nursing Knowledge”) OR (MH “Behavior”))		'Nurse attitude'/de OR ('nurse'/exp OR 'nursing role'/de) AND ('perception'/de OR 'attitude'/de OR 'awareness'/de OR 'knowledge'/exp OR 'beliefs'/de OR 'behavior'/de)	

^a^MeSH: Medical Subject Headings.

**Table 3:** Example of a search strategy in MEDLINE (PubMed); # represents the different sequences of the search.

#	Query	Search details
10	#3 AND #6 AND #9	(“Climate change*”[Title/Abstract] OR “global warming”[Title/Abstract] OR “climate crisis”[Title/Abstract] OR “climate warming”[Title/Abstract] OR “climate issue*”[Title/Abstract] OR “climate vulnerabilit*”[Title/Abstract] OR “climate emergenc*”[Title/Abstract] OR “Greenhouse Effect”[Title/Abstract] OR “climate action”[Title/Abstract] OR (“Climate Change”[MeSH^a^ Terms] OR “Greenhouse Effect”[MeSH Terms])) AND (“Nurse”[Title/Abstract] OR “Nurses”[Title/Abstract] OR “nursing”[Title/Abstract] OR (“Nurses”[MeSH Terms] OR “Nurse’s Role”[MeSH Terms])) AND (“attitude*”[Title/Abstract] OR “perception*”[Title/Abstract] OR “Awareness”[Title/Abstract] OR “perspective*”[Title/Abstract] OR “belief*”[Title/Abstract] OR “views”[Title/Abstract] OR “behavior*”[Title/Abstract] OR “opinion*”[Title/Abstract] OR “concern*”[Title/Abstract] OR “understanding”[Title/Abstract] OR “knowledge*”[Title/Abstract] OR (“Perception”[MeSH Terms:noexp] OR “Attitude”[MeSH Terms:noexp] OR “Attitude of Health Personnel”[MeSH Terms:noexp] OR “Awareness”[MeSH Terms] OR “Knowledge”[MeSH Terms] OR “Behavior”[MeSH Terms:noexp]))
9	#7 OR #8	“Attitude*” [Title/Abstract] OR “perception*” [Title/Abstract] OR “awareness” [Title/Abstract] OR “perspective*” [Title/Abstract] OR “belief*” [Title/Abstract] OR “views” [Title/Abstract] OR “behavior*” [Title/Abstract] OR “opinion*” [Title/Abstract] OR “concern*” [Title/Abstract] OR “understanding” [Title/Abstract] OR “knowledge*” [Title/Abstract] OR “Perception” [MeSH Terms:noexp] OR “Attitude” [MeSH Terms:noexp] OR “Attitude of Health Personnel” [MeSH Terms:noexp] OR “Awareness” [MeSH Terms] OR “Knowledge” [MeSH Terms] OR “Behavior” [MeSH Terms:noexp]
8	“Perception”[Mesh:NoExp] OR “Attitude”[Mesh:NoExp] OR “Attitude of Health Personnel”[Mesh:NoExp] OR “Awareness”[Mesh] OR “Knowledge”[Mesh] OR “Behavior”[Mesh:NoExp]	“Perception”[MeSH Terms:noexp] OR “Attitude”[MeSH Terms:noexp] OR “Attitude of Health Personnel”[MeSH Terms:noexp] OR “Awareness”[MeSH Terms] OR “Knowledge”[MeSH Terms] OR “Behavior”[MeSH Terms:noexp]
7	Attitude*[Title/Abstract] OR perception*[Title/Abstract] OR awareness[Title/Abstract] OR perspective*[Title/Abstract] OR belief*[Title/Abstract] OR views[Title/Abstract] OR behavior*[Title/Abstract] OR opinion*[Title/Abstract] OR concern*[Title/Abstract] OR understanding[Title/Abstract] OR knowledge*[Title/Abstract]	“Attitude*”[Title/Abstract] OR “perception*”[Title/Abstract] OR “awareness”[Title/Abstract] OR “perspective*”[Title/Abstract] OR “belief*”[Title/Abstract] OR “views”[Title/Abstract] OR “behavior*”[Title/Abstract] OR “opinion*”[Title/Abstract] OR “concern*”[Title/Abstract] OR “understanding”[Title/Abstract] OR “knowledge*”[Title/Abstract]
6	#4 OR #5	“Nurse”[Title/Abstract] OR “Nurses”[Title/Abstract] OR “nursing”[Title/Abstract] OR “Nurses”[MeSH Terms] OR “Nurse’s Role”[MeSH Terms]
5	“Nurses”[Mesh] OR “Nurse’s Role”[Mesh]	“Nurses”[MeSH Terms] OR “Nurse’s Role”[MeSH Terms]
4	Nurse[Title/Abstract] OR nurses[Title/Abstract] OR nursing[Title/Abstract]	“Nurse”[Title/Abstract] OR “nurses”[Title/Abstract] OR “nursing”[Title/Abstract]
3	#1 OR #2	“Climate change*”[Title/Abstract] OR “global warming”[Title/Abstract] OR “climate crisis”[Title/Abstract] OR “climate warming”[Title/Abstract] OR “climate issue*”[Title/Abstract] OR “climate vulnerabilit*”[Title/Abstract] OR “climate emergenc*”[Title/Abstract] OR “Greenhouse Effect”[Title/Abstract] OR “climate action”[Title/Abstract] OR “Climate Change”[MeSH Terms] OR “Greenhouse Effect”[MeSH Terms]
2	“Climate Change”[Mesh] OR “Greenhouse Effect”[Mesh]	“Climate Change”[MeSH Terms] OR “Greenhouse Effect”[MeSH Terms]
1	“Climate change*”[Title/Abstract] OR “global warming”[Title/Abstract] OR “climate crisis”[Title/Abstract] OR “climate warming”[Title/Abstract] OR “climate issue*”[Title/Abstract] OR “Climate vulnerabilit*”[Title/Abstract] OR “Climate Emergenc*”[Title/Abstract] OR “Greenhouse Effect”[Title/Abstract] OR “climate action”[Title/Abstract]	“Climate change*”[Title/Abstract] OR “global warming”[Title/Abstract] OR “climate crisis”[Title/Abstract] OR “climate warming”[Title/Abstract] OR “climate issue*”[Title/Abstract] OR “climate vulnerabilit*”[Title/Abstract] OR “climate emergenc*”[Title/Abstract] OR “Greenhouse Effect”[Title/Abstract] OR “climate action”[Title/Abstract]

^a^MeSH: Medical Subject Headings.

### Selection of Relevant and Reliable Studies

For this step, studies identified by the database search were first imported into the bibliographic management software EndNote and then transferred to Covidence (Veritas Health Innovation) to remove duplicates and facilitate the sorting and selection of relevant studies based on previously defined inclusion and exclusion criteria.

Once the duplicates were removed, an initial selection was begun based on the titles and abstracts, by 2 members of the research team (TD and AB) working independently. Studies focused on nurses’ perceptions of climate change will be retained. No date limits will be applied. All studies that address an environmental issue other than climate change, concern health professionals other than nurses, or are published in a language other than English or French will be excluded. Any disagreement regarding inclusion or exclusion will be resolved by consensus and, if necessary, by consultation with a third team member.

Once this initial stage of selection is completed, a second stage of selection will determine which articles will be included in the final analysis, based on their full text. This stage will be carried out by the same 2 researchers performing the initial selection. Studies whose full text is not available, studies that do not provide the authors’ names or publication dates, or those that constitute expert opinions or commentaries will be excluded. Accordingly, only empirical studies will be considered. Once again, any disagreements will be resolved by consensus between the 2 reviewers. A third reviewer will be consulted to establish consensus if the 2 reviewers’ opinions diverge.

The results of the selection process will be presented in a PRISMA (Preferred Reporting Items for Systematic Reviews and Meta-Analyses) diagram [27], showing the number of studies selected at each stage of selection.

### Charting the Data From the Included Studies

This step consists of extracting the data from the full-text articles selected. For this purpose, a Microsoft Excel table will be developed. It will include the following sections: title, authors, year of publication, geographic area, study objectives, methodology, and key findings.

### Collating, Summarizing, and Reporting the Findings

The data extracted in the previous step will be analyzed by the research team. This analysis may include an informed discussion based on an in-depth examination of the findings and directed toward answering the research question and fulfilling the study’s objective. Quantitative and qualitative data will be analyzed altogether and categorized according to the type of perception to which it refers (eg, perception of nurses’ role toward climate change or perception of their needs regarding climate change).

### Grey Literature Search

To supplement the results of the bibliographic database search, we plan to also conduct a search of the grey literature. To this end, we will consult the websites of associations and organizations related to nursing and those focused on the environment. Social media searches or reporting were not considered. We targeted nursing associations and organizations in the province of Québec, those at the federal level in Canada and the United States, and those at the international level. The following associations in Table 4 were identified through the scientific literature [18,28,29] and through a Google search using a combination of keywords related to nursing associations or organizations, climate change, and geographic area of interest (ie, Canada, United States, or international).

**Table 4:** Nursing associations and organizations identified for grey literature search.

Location and name of the association or organization		Website
**Province of Québec**		
	Quebec Nurses’ Association	[30]
	Ordre des infirmières et infirmiers du Québec	[31]
**Canada**		
	Canadian Nurses Association	[32]
	Canadian Association of Nurses for the Environment	[33]
	Canadian Federation of Nurses Unions	[34]
	Canadian Nurse Educator Institute	[35]
	Canadian Association of Schools of Nursing	[36]
**United States**		
	American Nurses Association	[37]
	American Association of Colleges of Nursing	[38]
	American Academy of Nursing	[39]
	Alliance of Nurses for Healthy Environments	[40]
	National Student Nurses’ Association	[41]
**International**		
	World Health Organization	[42]
	International Council of Nurses	[43]
	Secrétariat international des infirmières et des infirmiers de l'espace francophone	[44]
	Nurses Climate Challenge	[45]

Three approaches will be used to search the grey literature. The first approach will consist of searching by navigating the websites of the various associations and consulting their different sections, such as the section on publications; the second approach will involve using the search function of the websites to search for the keywords that were defined for the bibliographic database searches; finally, the third approach will consist of searching on Google by combining the name of the association with the keywords defined for the bibliographic database searches. The data will be extracted into an Excel spreadsheet that will contain the following sections: date of search, site searched, type of search performed (referencing one of the three approaches listed above), number of results found, number of results retained, and comments. For searches conducted by navigating the sites, the sections consulted will be specified, and for keywords searches, the terms or expressions used in searches will be specified.

## Results

The results will allow us, firstly, to ascertain how nurses perceive climate change; and secondly, to analyze these perceptions in relation to nurses’ contexts (personal or professional), the roles they can play, and what they require to better address this phenomenon.

The results will be gathered and shared according to the type of perception they refer to (eg, perception of climate change’s causes and impacts, perception of nurses’ role toward climate change, and perception of their needs regarding climate change).

We hope that the results will point toward targeted climate change awareness, raising interventions for nurses and guide new interventions aimed at improving nursing practice in a climate change context. In addition, with climatic hazards, such as extreme heat episodes and floods, expected to become increasingly frequent in coming years, a better understanding of climate issues among nurses will enable them to better assess risks and provide patients with appropriate advice and care. The results may also move forward engagement and dialogue on this important topic among nurses as well as raise awareness in nursing faculty to include these important issues in the education of future nurses. The findings will also open avenues for future research on the potential of nursing involvement in the mitigation, adaptation, and resilience of communities in the context of climate change.

## Discussion

### Expected Outcomes

To our knowledge, this study is the first exploratory review of the literature aiming to identify nurses’ perceptions of climate change. Its relevance comes from the fact that studies have been conducted in recent years that address nurses’ perceptions of climate change [16,17,21-23] but have not been compared and interpreted as a body of literature. The preliminary results of this exploratory literature review suggest that nurses could be preoccupied about climate change to a certain extent [20] but could lack knowledge about climate change, its causes, and its impacts on health [15,20,22]. The perceptions of their role in the fight against climate change as well as concrete ways to include the fight against climate change in their daily work despite constraints seems also unclear [17,22]. The full results of this review of the literature will allow us to cross-reference the different perceptions nurses have of climate change, as described in the various studies, and to develop an overall portrait of these perceptions. These results will allow us to identify ways to fill the possible gap between the importance of the nursing role in addressing the health impacts of climate change and the lack of knowledge and awareness of nurses in this matter. Therefore, this study should also help nursing organizations, educational institutions, and policy makers to better identify the elements that would help prepare these health care workers to face this climate crisis and the subsequent impacts on the health and well-being of the communities and populations they serve. Research could also help better identify promising strategies to empower nurses to fully carry out their advocacy role toward their patients who are the most vulnerable to climate change.

One of the strengths of the search strategy is that it targets diverse interdisciplinary databases in which articles from different fields, such as biomedical sciences and social sciences, are indexed. This will give us a broader view of the scope of the existing literature. In addition, this search is supplemented by a search of the grey literature, which will allow us to find relevant documents that cannot always be found in current bibliographic databases.

### Review Limitations

One of the limitations of this exploratory review of the literature is that it only includes publications in French and English, which means that all publications in other languages that may be relevant to the topic will not be taken into consideration. Nevertheless, as the vast majority of scientific publications are written in English, our findings should be of interest. Another limitation is that we do not plan to assess the quality of studies. However, this step is optional in the case of a scoping review [25].

### Conclusions

The full results will be disseminated through various means, such as international nursing conferences and articles, including an article in a nursing association journal. Authors will also publicize the published articles in their respective professional network. This dissemination strategy hopes to reach nurses and raise awareness toward their role in the fight against climate change and the ways to address its health effects.

## Abbreviations

PRISMA: Preferred Reporting Items for Systematic Reviews and Meta-Analyses
